# Cardiac index predicts long-term outcomes in patients with heart failure

**DOI:** 10.1371/journal.pone.0252833

**Published:** 2021-06-04

**Authors:** Tatsuro Ibe, Hiroshi Wada, Kenichi Sakakura, Yusuke Ugata, Hisataka Maki, Kei Yamamoto, Masaru Seguchi, Yousuke Taniguchi, Hiroyuki Jinnouchi, Hideo Fujita

**Affiliations:** Division of Cardiovascular Medicine, Saitama Medical Center, Jichi Medical University, Saitama, Japan; Universita degli Studi di Napoli Federico II, ITALY

## Abstract

**Background:**

The role of cardiac index (CI) and right atrial pressure (RAP) for predicting long-term outcomes of heart failure has not been well established. The aim of this study was to investigate long-term cardiac outcomes in patients with heart failure having various combinations of CI and RAP.

**Methods:**

A total of 787 heart failure patients who underwent right-heart catheterization were retrospectively categorized into the following four groups: Preserved CI (≥2.5 L/min/m^2^) and Low RAP (<8 mmHg) (PRE-CI/L-RAP; n = 285); Preserved CI (≥2.5 L/min/m^2^) and High RAP (≥8 mmHg) (PRE-CI/H-RAP; n = 242); Reduced CI (<2.5 L/min/m^2^) and Low RAP (<8 mmHg) (RED-CI/L-RAP; n = 123); and Reduced CI (<2.5 L/min/m^2^) and High RAP (≥8 mmHg) (RED-CI/H-RAP; n = 137). Survival analysis was applied to investigate which groups were associated with major adverse cardiovascular events (MACE).

**Results:**

The RED-CI/L-RAP and RED-CI/H-RAP groups were significantly associated with MACE as compared with the PRE-CI/L-RAP and PRE-CI/H-RAP groups after adjustment for confounding factors (RED-CI/L-RAP vs. PRE-CI/L-RAP: HR 2.11 [95% CI 1.33–3.37], *p* = 0.002; RED-CI/H-RAP vs. PRE-CI/L-RAP: HR 2.18 [95% CI 1.37–3.49], *p* = 0.001; RED-CI/L-RAP vs. PRE-CI/H-RAP: HR 1.86 [95% CI 1.16–3.00], *p* = 0.01; RED-CI/H-RAP vs. PRE-CI/H-RAP: HR 1.92 [95% CI 1.26–2.92], *p* = 0.002), whereas the difference between the RED-CI/H-RAP and RED-CI/L-RAP groups was not significant (HR 1.03 [95% CI 0.64–1.66], *p* = 0.89).

**Conclusions:**

The hemodynamic severity categorized by CI and RAP levels provided clear risk stratification in patients with symptomatic heart failure. Low CI was an independent predictor of long-term cardiac outcomes.

## Introduction

Right heart catheterization (RHC) is the gold standard for evaluating cardiac hemodynamics. The Forrester classification is a well-known index to stratify patients with heart failure by cardiac index (CI) and pulmonary artery wedge pressure (PAWP), both of which are associated with in-hospital mortality [1, 2]. Although the Forrester classification was originally established in patients with acute myocardial infarction [1, 2], the classification is mainly used to assess hemodynamics in patients with heart failure in contemporary clinical practice. The ESCAPE trial aimed to evaluate the efficacy of RHC-guided treatment for heart failure [3], but could not show the superiority of RHC-guided strategy over conventional strategy. However, the study subjects were limited to those patients with heart failure having reduced ejection fraction (HFrEF) [left ventricular ejection fraction (LVEF) ≤30%] in the ESCAPE trial. Although the Forrester classification and the results of ESCAPE trial considerably influenced our daily practice of heart failure, the efficacy of RHC for cardiac outcomes, regardless of etiology and left ventricular (LV) function, remains unclear. Moreover, the relationship between hemodynamic parameters and long-term cardiac outcomes in patients with heart failure has not been well established.

CI is a parameter of cardiac function reflecting not only left heart function but also right heart function. Furthermore, CI is a parameter related to both systolic and diastolic ventricular function [4]. RAP is an index of compensation reflecting right and left heart [5–7]. Combinations of CI and RAP would be complementary indices to indicate heart failure status. Therefore, combinations of CI and RAP could be better hemodynamic parameters to predict clinical outcomes in patients with heart failure. The aims of this study were to investigate the efficacy of various combinations of CI and RAP in providing clear risk stratification and the role of hemodynamic parameters in long-term cardiac outcomes.

## Methods

### Study design

We retrospectively reviewed patients admitted to our institute. The inclusion criteria were: (1) patients admitted for symptomatic heart failure [New York Heart Association (NYHA) functional classification ≥II and American College of Cardiology Foundation/American Heart Association (ACCF/AHA) classification Stage C or D], and (2) patients who underwent RHC at a compensated stage between January 2007 and December 2017. The exclusion criteria were: (1) patients with acute myocardial infarction; (2) patients with pre-capillary pulmonary hypertension (pulmonary hypertension categorized into groups 1, 3, 4, and 5); (3) patients with heart failure with constrictive pericarditis or congenital shunt disease; (4) patients with heart failure receiving hemodialysis; and (5) patients who had insufficient data for RHC. The study patients were divided into four groups according to the cut-off values of CI and RAP as reported in previous studies [8, 9]. Then, we categorized the four groups as follows: (i) Preserved CI and Low RAP group (PRE-CI/L-RAP), CI ≥2.5 L/min/m^2^ and RAP <8 mmHg; (ii) Preserved CI and High RAP group (PRE-CI/H-RAP), CI ≥2.5 L/min/m^2^ and RAP ≥8 mmHg; (iii) Reduced CI and Low RAP group (RED-CI/L-RAP), CI <2.5 L/min/m^2^ and RAP <8 mmHg; and (iv) Reduced CI and High RAP group (RED-CI/H-RAP), CI <2.5 L/min/m^2^ and RAP ≥8 mmHg. The study was approved by the institutional review board at Saitama Medical Center, Jichi Medical University (S20-014), and written informed consent was waived because of the retrospective design of the study.

### Follow-up

Clinical follow-up was performed via office visits and medical records. The follow-up period was until December 2018. The day when RHC was performed was defined as the index day. The primary endpoint was major adverse cardiovascular events (MACE), defined as the composite of cardiac death, re-admission due to heart failure, and left ventricular assist device (LVAD) implantation. The day of either cardiac death, first re-admission due to heart failure, or LVAD implantation was considered as an event day.

### Right heart catheterization

In these study subjects, RHC was performed at a compensated stage for symptoms of heart failure [10]. An external pressure transducer was zeroed at the mid-thoracic line with the patient in the supine position [11]. The average of several consecutive pressure waves over 9 seconds was recorded as the pressure measurement value during RHC [10]. Cardiac output (CO) was measured using thermodilution with cold saline infusion.

### Definition of clinical characteristics

Left ventricular (LV) systolic function was categorized as reduced LVEF (LVEF <40%), mid-range LVEF (40%≤ LVEF <50%), or preserved LVEF (LVEF ≥50%) by echocardiographic findings [12]. Hypertension was defined as a past medical history of hypertension or medical treatment for hypertension before admission [13]. Diabetes mellitus was defined as a hemoglobin A1c level ≥6.5% or treatment for diabetes mellitus before admission [13]. Hyperlipidemia was defined as a low-density lipoprotein cholesterol level ≥140 mg/dL or treatment for hyperlipidemia before admission [13]. Hyperuricemia was defined as a uric acid level >7.0 mg/dL or treatment for hyperuricemia before admission [14]. Anemia was defined as a hemoglobin level <13 g/dL for men and <12 g/dL for women [15]. Renal function was evaluated by the estimated glomerular filtration rate (eGFR) using the Modification of Diet in Renal Disease formula modified for the Japanese population [16]. Impaired renal function was defined as eGFR<60 mL/min/1.73 m^2^ [13]. Estimated right ventricular systolic pressure (eRVSP) measured by echocardiography was calculated as the sum of the peak RV-right atrium (RA) gradient, while RA pressure was estimated by the diameter and respiratory change of the inferior vena cava, as reported previously [17].

### Statistical analysis

Continuous variables are expressed as mean ± standard deviation (SD). Analysis of normally or non-normally distributed continuous variables was performed using the Shapiro-Wilk test. Non-parametric continuous variables were analyzed using the Kruskal-Wallis test. Categorical variables were expressed as frequencies and percentages, and analyzed via the chi-square test. Survival analyses were carried out using the Kaplan-Meier method, and the curves were then compared using the log-rank test. Multivariate Cox hazard analysis was also applied to investigate whether each group predicted MACE after adjustment for confounding factors for heart failure and other hemodynamic parameters (age [18], male sex [19], overweight [20], anemia [21], atrial fibrillation or flutter [22], hyperuricemia [23], impaired renal function [24], ischemic heart disease [25], LVEF [26–29], use of loop diuretics [30], mean pulmonary artery pressure, and PAWP). The statistical analyses were performed using SPSS 19/Windows statistical software (SPSS Inc, Chicago, IL, USA).

## Results

**Fig 1:**
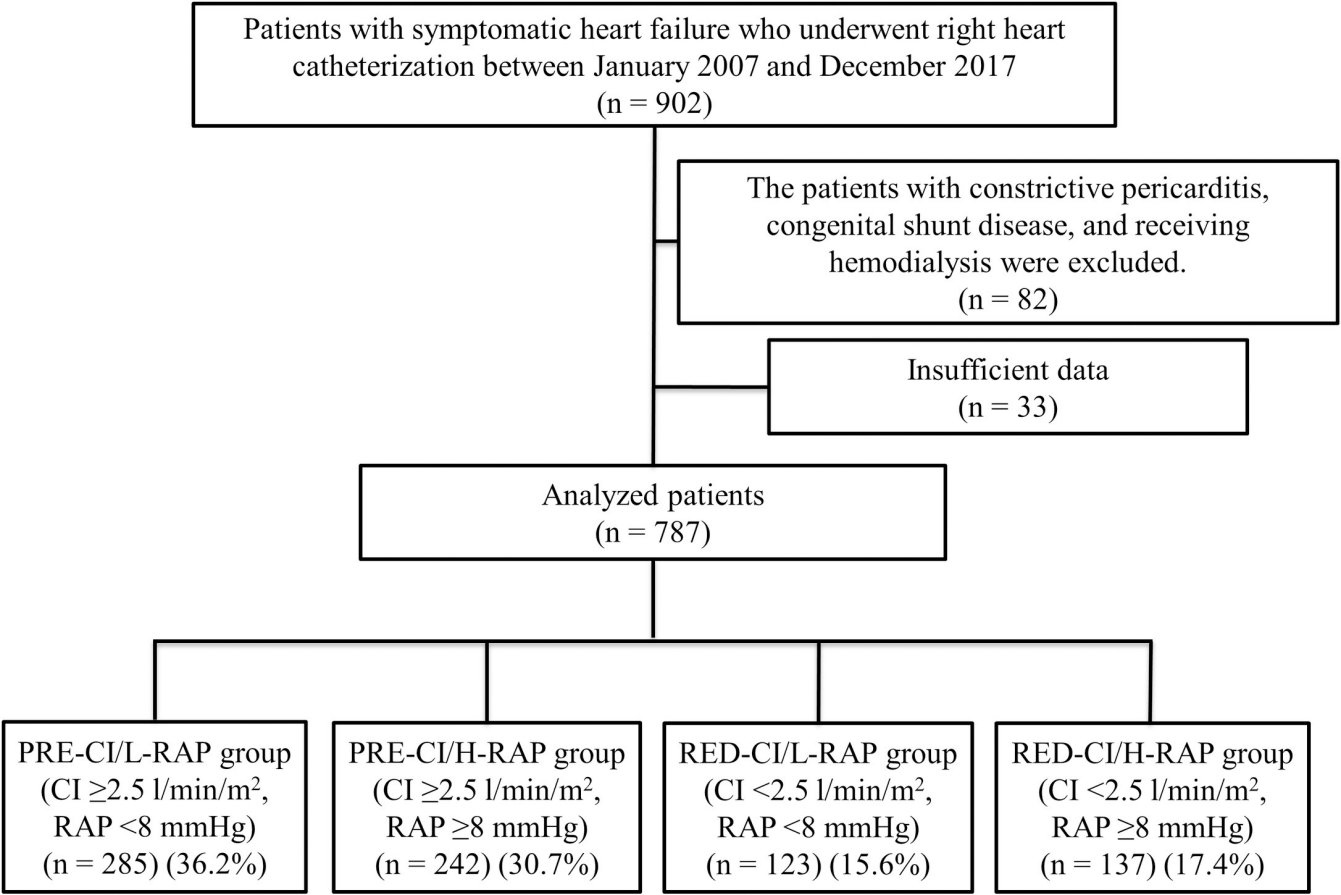
Patient enrollment. CI, cardiac index; RAP, right atrial pressure.

From January 2007 to December 2017, a total of 902 patients were admitted to our hospital for symptomatic heart failure and underwent RHC during their hospitalization. Eighty-two patients were excluded because of underlying diseases such as constrictive pericarditis, congenital shunt disease, or requirement for hemodialysis. Thirty-three patients who had insufficient data for RHC were also excluded from the study. The remaining 787 patients with symptomatic heart failure were included as the final study population. Based on their values of CI and RAP, the study patients were categorized into the four groups PRE-CI/L-RAP (n = 285), PRE-CI/H-RAP (n = 242), RED-CI/L-RAP (n = 123), and RED-CI/H-RAP (n = 137) (Fig 1). The median follow-up period was 22 months.

The clinical characteristics of the study cohort are described in Table 1. LV systolic function (reduced, mid-range, or preserved LVEF) was significantly different among the four groups (*p* < 0.001). Reduced LVEF was most common in the RED-CI/H-RAP group, followed by the RED-CI/L-RAP and PRE-CI/L-RAP groups, and least common in the PRE-CI/H-RAP group. There were no significant differences in the etiology of heart failure among the 4 groups. The levels of BNP were highest in the RED-CI/H-RAP group, followed by the RED-CI/L-RAP and PRE-CI/L-RAP groups, and lowest in the PRE-CI/H-RAP group. Over 70% of patients received beta-blockers and angiotensin-converting enzyme inhibitors or angiotensin receptor blockers, and over 80% of patients with reduced CI had those medications. Although many subjects in this study had loop diuretics, the prevalence of diuretic usage was significantly different among the 4 groups (*p* = 0.001). The parameters of RHC are described in Table 2. All the hemodynamic parameters such as systolic pulmonary artery pressure, mean pulmonary artery pressure (mPAP), diastolic pulmonary artery pressure, PAWP, CO, heart rate, and pulmonary vascular resistance were significantly different among the 4 groups. The levels of PAWP were higher in the RAP ≥8 mmHg groups than in the RAP <8 mmHg groups.

**Table 1:** Clinical characteristics.

	PRE-CI/L-RAP (n = 285)	PRE-CI/H-RAP (n = 242)	RED-CI/L-RAP (n = 123)	RED-CI/H-RAP (n = 137)	*P* value
Age (years)	66.3 ± 12.4	63.5 ± 15.1	64.0 ± 13.6	62.5 ± 14.1	0.04
Male, n (%)	182 (63.9%)	153 (63.2%)	83 (67.5%)	112 (81.8%)	0.001
BMI (kg/m^2^)	23.2 ± 4.1	25.8 ± 5.6	23.6 ± 5.1	25.5 ± 5.4	<0.001
Heart rate at admission (beat/min)	90.2 ± 26.4 (n = 284)	90.0 ± 27.0	92.8 ± 28.8 (n = 122)	98.3 ± 30.1	0.06
Systolic blood pressure at admission (mmHg)	131.9 ± 30.7	132.3 ± 30.1	128.3 ± 29.6	123.3 ± 22.9	0.05
Left ventricular systolic function					
Reduced LVEF, n (%)	135 (47.4%)	107 (44.2%)	77 (62.6%)	93 (67.9%)	<0.001
Mid-range LVEF, n (%)	45 (15.8%)	25 (10.3%)	15 (12.2%)	17 (12.4%)	
Preserved LVEF, n (%)	105 (36.8%)	110 (45.5%)	31 (25.2%)	27 (19.7%)	
Principal etiology of heart failure					
Ischemic heart disease, n (%)	35 (12.3%)	26 (10.7%)	12 (9.8%)	20 (14.6%)	0.90
Valvular heart disease, n (%)	67 (23.5%)	63 (26.0%)	27 (22.0%)	27 (19.7%)	
Cardiomyopathy, n (%)	26 (9.1%)	18 (7.4%)	11 (8.9%)	13 (9.5%)	
Others or unknown, n (%)	157 (55.1%)	135 (55.8%)	73 (59.3%)	77 (56.2%)	
Comorbidities					
Hypertension, n (%)	144 (50.5%)	137 (56.6%)	60 (48.8%)	73 (53.3%)	0.42
Diabetes mellitus, n (%)	79 (27.7%)	95 (39.3%)	48 (39.0%)	53 (38.7%)	0.02
Hyperlipidemia, n (%)	115 (40.4%)	101 (41.7%)	63 (51.2%)	67 (48.9%)	0.11
Hyperuricemia, n (%)	125 (43.9%)	130 (53.7%)	77 (62.6%)	92 (67.2%)	<0.001
COPD, n (%)	10 (3.5%)	5 (2.1%)	3 (2.4%)	3 (2.2%)	0.74
Anemia, n (%)	100 (35.1%)	95 (39.3%)	24 (19.5%)	30 (21.9%)	<0.001
Impaired renal function (eGFR <60 mL/min/1.73 m^2^), n (%)	126 (44.2%)	133 (55.0%)	65 (52.8%)	69 (50.4%)	0.09
Atrial fibrillation or flutter, n (%)	95 (33.3%)	90 (37.2%)	67 (54.5%)	76 (55.5%)	<0.001
Echocardiographic characteristics					
LAD (mm)	49.5 ± 9.0 (n = 283)	52.2 ± 8.9 (n = 235)	51.3 ± 7.8 (n = 122)	52.7 ± 9.5 (n = 135)	0.004
LVDd (mm)	58.6 ± 10.2 (n = 283)	58.7 ± 11.9 (n = 235)	60.1 ± 10.6 (n = 122)	61.5 ± 10.5 (n = 135)	0.02
LVDs (mm)	45.8 ± 12.6 (n = 282)	45.2 ± 14.6 (n = 235)	49.0 ± 12.6 (n = 122)	50.9 ± 12.3 (n = 135)	<0.001
LVEF (%)	43.0 ± 17.6 (n = 282)	45.5 ± 19.2 (n = 236)	37.1 ± 16.3 (n = 122)	35.1 ± 16.0 (n = 135)	<0.001
eRVSP (mmHg)	35.3 ± 16.6 (n = 265)	40.9 ± 16.9 (n = 226)	36.7 ± 18.7 (n = 120)	40.3 ± 14.7 (n = 134)	<0.001
Laboratory data					
Hemoglobin (g/dL)	13.2 ± 2.1	12.8 ± 2.3	14.1 ± 2.0	14.2 ± 1.9	<0.001
Na (mEq/L)	139.5 ± 3.4	139.7 ± 3.2	139.3 ± 3.0	138.7 ± 4.0	0.20
K (mEq/L)	4.3 ± 0.5	4.3 ± 0.5	4.3 ± 0.5	4.3 ± 0.5	0.27
eGFR (mL/min/1.73 m^2^)	63.6 ± 23.0	57.6 ± 22.0	58.3 ± 19.5	57.7 ± 17.3	0.008
Uric acid (mg/dL)	6.8 ± 2.3 (n = 284)	7.2 ± 2.2 (n = 240)	7.6 ± 2.2	8.0 ± 2.4 (n = 136)	<0.001
BNP (pg/mL)	783.3 ± 983.8 (n = 280)	665.7 ± 767.7 (n = 234)	948.5 ± 953.6 (n = 122)	1014.6 ± 1010.4 (n = 136)	<0.001
Medications					
Angiotensin-converting enzyme inhibitor, n (%)	152 (53.3%)	114 (47.1%)	67 (54.5%)	93 (67.9%)	0.002
Angiotensin receptor blocker, n (%)	79 (27.7%)	63 (26.0%)	36 (29.3%)	19 (13.9%)	0.009
Beta-blocker, n (%)	230 (80.7%)	179 (74.0%)	112 (91.1%)	123 (89.8%)	<0.001
Calcium channel blocker, n (%)	54 (18.9%)	79 (32.6%)	19 (15.4%)	18 (13.1%)	<0.001
Loop diuretics, n (%)	223 (78.2%)	213 (88.0%)	108 (87.8%)	125 (91.2%)	0.001
Thiazide diuretics, n (%)	11 (3.9%)	10 (4.1%)	3 (2.4%)	6 (4.4%)	0.84
Mineralocorticoid receptor antagonist, n (%)	140 (49.1%)	111 (45.9%)	74 (60.2%)	89 (65.0%)	0.001
Digitalis, n (%)	14 (4.9%)	15 (6.2%)	11 (8.9%)	6 (4.4%)	0.36
Oral inotropic agent, n (%)	2 (0.7%)	3 (1.2%)	0	1 (0.7%)	0.64
Statin, n (%)	106 (37.2%)	94 (38.8%)	58 (47.2%)	53 (38.7%)	0.29
Amiodarone, n (%)	22 (7.7%)	12 (5.0%)	11 (8.9%)	16 (11.7%)	0.12

BMI, body mass index; LVEF, left ventricular ejection fraction; COPD, chronic obstructive pulmonary disease; eGFR, estimated glomerular filtration rate; LAD, left atrium dimension; LVDd, left ventricular diastolic dimension; LVDs, left ventricular systolic dimension; eRVSP, estimated right ventricular systolic pressure; BNP, brain natriuretic peptide.

**Table 2:** Parameters of right heart catheterization.

	PRE-CI/L-RAP (n = 285)	PRE-CI/H-RAP (n = 242)	RED-CI/L-RAP (n = 123)	RED-CI/H-RAP (n = 137)	*P* value
RAP (mmHg)	4.9 ± 1.6	11.4 ± 3.8	4.9 ± 1.6	12.5 ± 4.2	<0.001
sPAP (mmHg)	31.7 ± 10.1	42.0 ± 12.7	30.2 ± 8.2	43.7 ± 13.1	<0.001
mPAP (mmHg)	20.1 ± 6.8	28.9 ± 8.4	20.0 ± 5.6	31.9 ± 9.2	<0.001
dPAP (mmHg)	13.1 ± 4.9	20.3 ± 6.5	13.5 ± 4.6	23.8 ± 7.3	<0.001
PAWP (mmHg)	12.1 ± 5.4	19.7 ± 6.6	12.5 ± 5.1	22.8 ± 7.0	<0.001
CO (L/min)	5.1 ± 1.1	5.6 ± 1.3	3.6 ± 0.6	3.8 ± 0.8	<0.001
CI (L/min/m^2^)	3.2 ± 0.6	3.3 ± 0.7	2.2 ± 0.3	2.1 ± 0.3	<0.001
Heart rate (beats/min)	72.5 ± 14.8 (n = 278)	73.6 ± 16.2 (n = 240)	72.8 ± 16.0 (n = 121)	79.1 ± 17.5	0.001
PVR (Wood units)	1.6 ± 0.8	1.7 ± 0.9	2.1 ± 1.0	2.5 ± 1.4	<0.001

RAP, right atrial pressure; sPAP, systolic pulmonary artery pressure; mPAP, mean pulmonary artery pressure; dPAP, diastolic pulmonary artery pressure; PAWP, pulmonary artery wedge pressure; CO, cardiac output; CI, cardiac index; PVR, pulmonary vascular resistance.

We additionally compared the parameters of echocardiography and RHC among reduced LVEF, mid-range LVEF, and preserved LVEF groups (Table 3). Left ventricular diastolic and systolic dimensions were significantly different among the three groups (*p* < 0.001), with the largest in the reduced LVEF group, followed by the mid-range LVEF group, and the smallest in the preserved LVEF group. Tricuspid annular plane systolic excursion and CI showed statistically significant differences among the three groups (*p* < 0.001), with the highest in the preserved LVEF group, followed by the mid-range group, and the lowest in the reduced LVEF group. Overall, the mid-range LVEF group was intermediate between the reduced and preserved LVEF groups regarding morphological findings and function. There were no significant differences in the values of mPAP (*p* = 0.09).

**Table 3:** Parameters of echocardiography and right heart catheterization of the groups stratified by LVEF.

	Reduced LVEF (n = 412)	Mid-range LVEF (n = 102)	Preserved LVEF (n = 273)	*P* value
Parameters of echocardiography				
LAD (mm)	50.8 ± 8.0 (n = 405)	50.0 ± 7.9 (n = 101)	52.1 ± 10.5 (n = 269)	0.10
LVDd (mm)	65.3 ± 9.0 (n = 405)	57.2 ± 7.8 (n = 101)	51.2 ± 8.6 (n = 269)	<0.001
LVDs (mm)	56.7 ± 9.0 (n = 404)	44.2 ± 6.6 (n = 101)	33.6 ± 7.1 (n = 269)	<0.001
LVEF (%)	26.5 ± 7.7 (n = 404)	44.7 ± 3.5	62.6 ± 7.8 (n = 269)	<0.001
eRVSP (mmHg)	36.9 ± 16.1 (n = 391)	36.0 ± 14.8 (n = 99)	40.7 ± 18.5 (n = 255)	0.02
E/e′	19.9 ± 8.3 (n = 330)	19.7 ± 10.4 (n = 81)	20.2 ± 9.0 (n = 197)	0.59
TAPSE (mm)	15.0 ± 4.1 (n = 197)	17.9 ± 5.0 (n = 36)	18.3 ± 5.6 (n = 97)	<0.001
Parameters of right heart catheterization				
RAP (mmHg)	8.3 ± 4.6	7.8 ± 4.8	8.3 ± 4.5	0.33
sRVP (mmHg)	36.6 ± 12.1 (n = 411)	35.5 ± 12.6 (n = 101)	38.7 ± 12.9	0.008
RVEDP (mmHg)	9.6 ± 5.2 (n = 410)	9.2 ± 5.4 (n = 101)	9.3 ± 4.4 (n = 271)	0.39
sPAP (mmHg)	36.8 ± 12.7	35.6 ± 13.2	37.0 ± 12.3	0.21
mPAP (mmHg)	25.5 ± 9.6	23.6 ± 8.9	24.3 ± 8.3	0.09
dPAP (mmHg)	18.4 ± 7.8	16.3 ± 7.2	15.9 ± 6.0	<0.001
PAWP (mmHg)	17.0 ± 7.8	15.6 ± 7.3	15.8 ± 6.9	0.13
CO (L/min)	4.7 ± 1.3	4.8 ± 1.4	5.0 ± 1.4	0.02
CI (L/min/m^2^)	2.7 ± 0.7	2.8 ± 0.7	3.1 ± 0.8	<0.001
Heart rate (beats/min)	76.0 ± 16.4 (n = 404)	74.4 ± 16.4 (n = 100)	71.1 ± 15.0 (n = 272)	0.001
PVR (Wood units)	1.9 ± 1.1	1.8 ± 1.1	1.8 ± 1.0	0.17

LVEF, left ventricular ejection fraction; LAD, left atrium dimension; LVDd, left ventricular diastolic dimension; LVDs, left ventricular systolic dimension; eRVSP, estimated right ventricular systolic pressure; E/e′, ratio of early diastolic mitral flow velocity / mitral tissue Doppler lengthening velocity; TAPSE, tricuspid annular plane systolic excursion; RAP, right atrial pressure; sRVP, systolic right ventricular pressure; RVEDP, right ventricular end-diastolic pressure; sPAP, systolic pulmonary artery pressure; mPAP, mean pulmonary artery pressure; dPAP, diastolic pulmonary artery pressure; PAWP, pulmonary artery wedge pressure; CO, cardiac output; CI, cardiac index; PVR, pulmonary vascular resistance.

**Fig 2:**
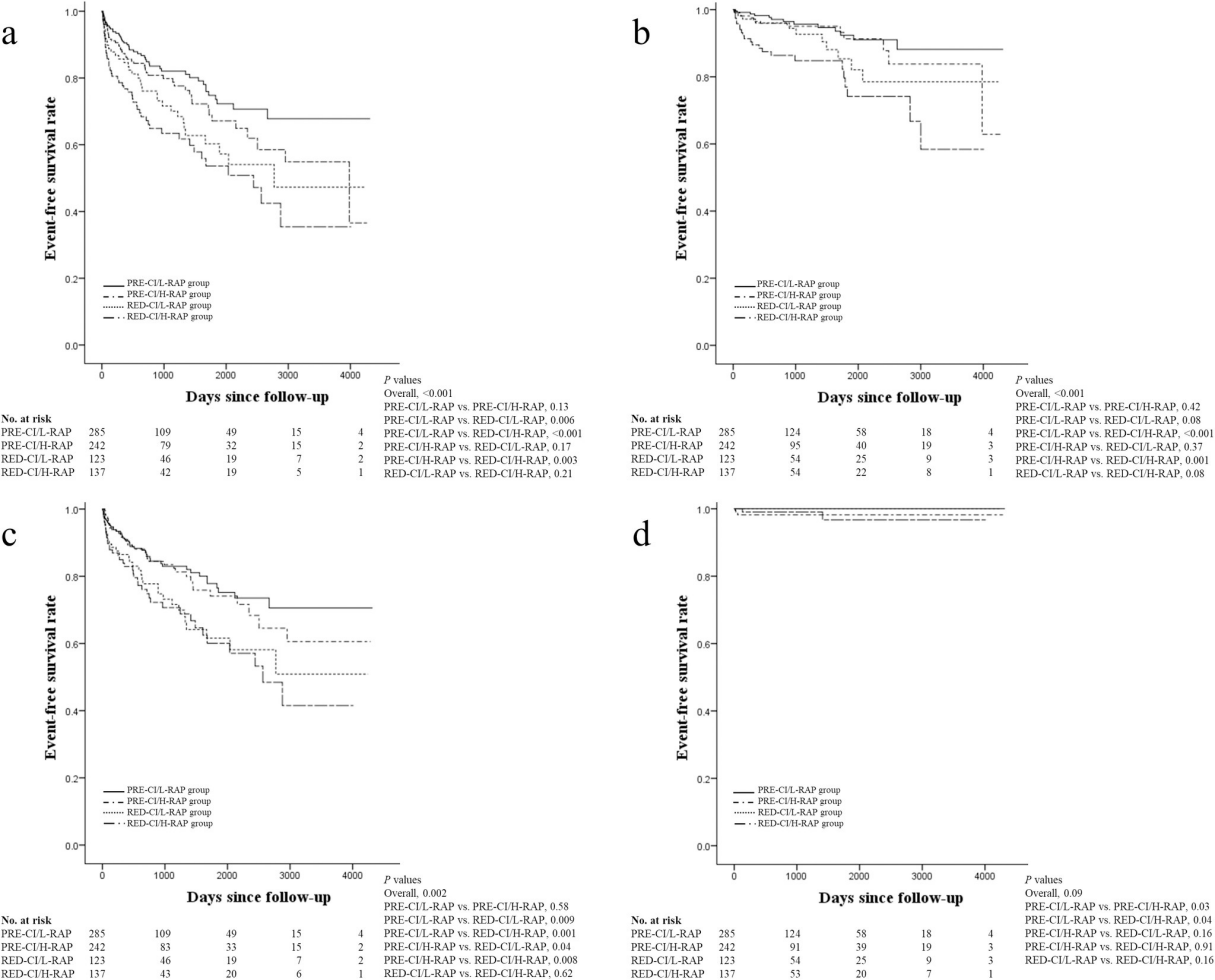
Kaplan-Meier curves for (a) major adverse cardiovascular events, (b) cardiac death, (c) readmission due to heart failure, and (d) implantation of a left ventricular assist device in the four groups. Comparison of the survival curves was performed using the log-rank test.

During the follow-up period, cardiac death, heart failure readmission, or LVAD implantation occurred in 60, 153, and 6 patients, respectively. As a result, the primary endpoints were observed in 181 patients. The Kaplan-Meier curves for the primary endpoints are shown in Fig 2A. Survival curves showed clear risk stratification in the order of hemodynamic severity. The log-rank test revealed a significant increase in adverse events in the RED-CI/L-RAP and RED-CI/H-RAP groups compared with that in the PRE-CI/L-RAP group (*p* = 0.006 for PRE-CI/L-RAP vs. RED-CI/L-RAP; *p* < 0.001 for PRE-CI/L-RAP vs. RED-CI/H-RAP), while there was no significant difference between the PRE-CI/L-RAP and PRE-CI/H-RAP groups (*p* = 0.13). The Kaplan-Meier curves for cardiac death, heart failure readmission, and LVAD implantation are shown in Fig 2B, 2C, and 2D. Survival curves for cardiac death and heart failure readmission also showed clear risk stratification, whereas those of LVAD implantation did not show statistical significance. As for survival curves of heart failure readmission, there were significant increases in events in the groups with CI <2.5 L/min/m^2^ (RED-CI/L-RAP and RED-CI/H-RAP) compared to the groups with CI ≥2.5 L/min/m^2^ (PRE-CI/L-RAP and PRE-CI/H-RAP) (Fig 2C). We made ROC curves for CI and RA separately to evaluate the discriminating ability of CI and RA for clinical outcomes in each group (S1 Fig). Neither CI nor RA had a discriminating ability for primary endpoints in any group.

The multivariate Cox hazard analysis also confirmed a significant increase in MACE in the RED-CI/L-RAP and RED-CI/H-RAP groups compared with the PRE-CI/L-RAP group even after adjustment for confounding factors (RED-CI/L-RAP, HR 2.11 [95% CI 1.33–3.37], *p* = 0.002; RED-CI/H-RAP, HR 2.18 [95% CI 1.37–3.49], *p* = 0.001) (Table 4, Model 1). Adjusted hazard ratios for the RED-CI/L-RAP and RED-CI/H-RAP groups, with the PRE-CI/H-RAP group as the reference, also showed a significant association of MACE (RED-CI/L-RAP, HR 1.86 [95% CI 1.16–3.00], *p* = 0.01; RED-CI/H-RAP, HR 1.92 [95% CI 1.26–2.92], *p* = 0.002) (Table 4, Model 2), whereas that of the RED-CI/H-RAP group, with the RED-CI/L-RAP group as the reference, had no statistical significance (HR 1.03 [95% CI 0.64–1.66], *p* = 0.89) (Table 4, Model 3). Multivariate Cox regression analysis predicting cardiac death did not show statistical significance between the PRE-CI/H-RAP and RED-CI/L-RAP groups (RED-CI/L-RAP group, HR 1.94 [95% CI 0.82–4.60], *p* = 0.13) (S1 Table), while that of heart failure readmission showed a significant increase in the RED-CI/L-RAP group compared with the PRE-CI/H-RAP group (HR 2.26 [95% CI 1.35–3.79], *p* = 0.002) (S2 Table). Elevated PAWP (PAWP ≥18 mmHg) showed no statistical significance for any events (Table 4, S1 and S2 Tables).

**Table 4:** Multivariate Cox regression analysis predicting primary endpoint.

Variables	Model 1			Model 2			Model 3		
	HR	95% CI	*P* value	HR	95% CI	*P* value	HR	95% CI	*P* value
Hemodynamic categorization of heart failure									
PRE-CI/H-RAP (vs. PRE-CI/L-RAP)	1.14	0.74–1.75	0.56						
RED-CI/L-RAP (vs. PRE-CI/L-RAP)	2.11	1.33–3.37	0.002						
RED-CI/H-RAP (vs. PRE-CI/L-RAP)	2.18	1.37–3.49	0.001						
Hemodynamic categorization of heart failure									
PRE-CI/L-RAP (vs. PRE-CI/H-RAP)				0.88	0.57–1.36	0.56			
RED-CI/L-RAP (vs. PRE-CI/H-RAP)				1.86	1.16–3.00	0.01			
RED-CI/H-RAP (vs. PRE-CI/H-RAP)				1.92	1.26–2.92	0.002			
Hemodynamic categorization of heart failure									
PRE-CI/L-RAP (vs. RED-CI/L-RAP)							0.47	0.30–0.75	0.02
PRE-CI/H-RAP (vs. RED-CI/L-RAP)							0.54	0.33–0.87	0.01
RED-CI/H-RAP (vs. RED-CI/L-RAP)							1.03	0.64–1.66	0.89
mPAP >20 mmHg	1.22	0.80–1.88	0.36	1.22	0.80–1.88	0.36	1.22	0.80–1.88	0.36
PAWP ≥18 mmHg	1.32	0.90–1.95	0.16	1.32	0.90–1.95	0.16	1.32	0.90–1.95	0.16
Age (10 year increase)	1.17	1.01–1.34	0.03	1.17	1.01–1.34	0.03	1.17	1.01–1.34	0.03
Male sex (vs. female)	0.82	0.58–1.14	0.24	0.82	0.58–1.14	0.24	0.82	0.58–1.14	0.24
Overweight (BMI ≥25 kg/m^2^)	0.93	0.67–1.30	0.68	0.93	0.67–1.30	0.68	0.93	0.67–1.30	0.68
Anemia	1.64	1.18–2.29	0.003	1.64	1.18–2.29	0.003	1.64	1.18–2.29	0.003
Atrial fibrillation or flutter	0.98	0.71–1.35	0.89	0.98	0.71–1.35	0.89	0.98	0.71–1.35	0.89
Hyperuricemia	0.89	0.63–1.26	0.53	0.89	0.63–1.26	0.53	0.89	0.63–1.26	0.53
Impaired renal function	1.10	0.79–1.53	0.57	1.10	0.79–1.53	0.57	1.10	0.79–1.53	0.57
Ischemic heart disease	1.80	1.22–2.67	0.003	1.80	1.22–2.67	0.003	1.80	1.22–2.67	0.003
Loop diuretic use	0.96	0.61–1.49	0.84	0.96	0.61–1.49	0.84	0.96	0.61–1.49	0.84
Categorization of LVEF									
Reduced LVEF (vs. preserved LVEF)	1.41	0.97–2.07	0.08	1.41	0.97–2.07	0.08	1.41	0.97–2.07	0.08
Mid-range LVEF (vs. preserved LVEF)	0.72	0.40–1.29	0.27	0.72	0.40–1.29	0.27	0.72	0.40–1.29	0.27

HR, hazard ratio; CI, confidence interval; mPAP, mean pulmonary artery pressure; PAWP, pulmonary artery wedge pressure; BMI, body mass index; LVEF, left ventricular ejection fraction.

## Discussion

The present study included 787 patients with symptomatic heart failure who underwent RHC. We investigated whether combinations of CI and RAP levels at a compensated stage could provide risk stratification for long-term cardiac outcomes. The major findings of this study were as follows: (i) Combinations of CI and RAP levels clearly stratified long-term cardiac outcomes of patients with symptomatic heart failure in the order of hemodynamic severity; (ii) Low CI (CI <2.5 L/min/m^2^) was associated significantly with MACE even after adjustment for clinically relevant confounding factors, whereas elevated RAP was not associated with MACE.

Previously, Cooper et al. investigated the relationship between hemodynamic parameters and mid-term clinical outcomes in ESCAPE trial subjects [31]. The study categorized patients with heart failure by CI and PAWP, which stratified mid-term (6 months) clinical outcomes. The predictors of post-discharge outcomes were elevated PAWP and RAP, whereas the levels of CI did not affect cardiac outcomes [31], which is opposite to our results. The ESCAPE trial limited the study subjects to the patients with HFrEF (LVEF ≤30%), and the follow-up period of the ESCAPE trial (6 months) was shorter than our study (median 22 months) [3]. Moreover, medications for heart failure in the ESCAPE trial were different from our study to some extent. For example, patients taking beta-blockers composed only 62% of the study subjects in the ESCAPE trial [3]. These differences may account for the above discordance. Cooper et al. and our study revealed that both combinations of CI and PAWP or RAP could stratify outcomes of heart failure. However, our results showed that cardiac function was preferable to volume overload for predicting cardiac outcomes. Patel et al. showed that low CI was independently associated with poor cardiac outcomes in patients with heart failure, although the study population was small (n = 187) [32]. The result of Patel et al. supports our study findings.

In our study, the group with CI <2.5 L/min/m^2^ and RAP ≥8 mmHg was significantly worse than the groups with CI ≥2.5 L/min/m^2^ for all outcomes except for LVAD implantation. Because the number of LVAD implantations was small in our study, the survival curves for LVAD implantation did not show statistical significance. The group with CI <2.5 L/min/m^2^ and RAP ≥8 mmHg is considered to be similar to Forrester subset 4 or Nohria-Stevenson classification Profile C (wet-cold) [1, 2, 33]. It is not surprising that the RED-CI/H-RAP group showed the worst clinical outcomes as reported in previous studies [1, 2, 33]. Our results revealed that the combinations of CI and RAP levels could predict long-term clinical outcomes like the combinations of CI and PAWP levels as Forrester et al. established [1, 2]. As for the comparison between the PRE-CI/H-RAP group and the RED-CI/L-RAP group, the RED-CI/L-RAP group was significantly associated with MACE compared with the PRE-CI/H-RAP group, which indicated that low CI was more closely associated with MACE than high RAP. Moreover, there was no statistical significance in events between low RAP and high RAP when the CI categories were the same. The above results mean that the groups with CI <2.5 L/min/m^2^ may predict MACE regardless of the levels of RAP. Increased RAP leads to venous congestion, which impairs organ function such as renal function [34]. As a result, the levels of RAP seem to affect heart failure readmission. However, our results showed that the levels of RAP did not indicate risk stratification for cardiac outcomes. RAP is a parameter with considerable fluctuation and is more easily affected by volume overload than CI. Although our study subjects underwent RHC at a compensated stage, there may be some subjects who needed more decongestion. The above bias might make RAP a less predictive parameter than CI. On the other hand, because cardiac function and filling pressure are interactive, decreased cardiac function causes volume overload, and volume overload leads to decreased cardiac function in patients with heart failure [35], which confirms that the prognosis of heart failure is mainly determined by cardiac function. CI would be a better predictor of long-term outcomes of heart failure compared with RAP. Furthermore, although CI was the lowest in the reduced LVEF group (Table 3), LVEF was not a predictor for adverse outcomes even after adjustment for confounding factors (Table 4, S1 and S2 Tables), which may indicate that low CI is a predictor of outcomes regardless of LVEF.

Clinical implications of the present study should be noted. Contemporary understanding of the relationship between hemodynamic parameters and outcomes is mainly based on the data before over one decade [1–3, 31, 36]. Regarding the investigation among study subjects who underwent therapy for heart failure in the present era, we revealed that hemodynamic severity at a compensated stage of heart failure could stratify long-term cardiac outcomes. Notably, CI was an independent predictor affecting long-term cardiac outcomes in patients with heart failure. The results of RHC tend to be used to manage volume status. However, our results suggest that reduced CI is the sign to reconsider optimum therapies, and we should consider advanced therapies for heart failure according to the results of CI rather than RAP.

### Study limitations

The study was a retrospective design in a single tertiary center, which resulted in significant selection bias. It is also possible that incomplete follow-up occurred, because clinical follow-up was performed via office visits or review of medical records. We must consider that the severity of heart failure in our study cohort might be mild to moderate, because the patients who needed LVAD implantation and heart transplantation were fewer than in previous studies [31, 36]. We did not investigate the impact of recovery of cardiac function on clinical outcome, because our study did not mention follow-up RHC. Regarding the impact of temporal hemodynamic change, further prospective studies that include follow-up of hemodynamic parameters are warranted.

## Conclusions

The categorization by CI and RAP values provides clear risk stratification in symptomatic heart failure. Notably, low CI is an independent predictor of worse cardiac outcomes.

## Supporting information

S1 Table Multivariate Cox regression analysis predicting cardiac death. (DOCX)

S2 Table Multivariate Cox regression analysis predicting heart failure readmission. (DOCX)

S1 Fig Receiver operating characteristic curves of cardiac index and right atrial pressure for the primary endpoint. CI, cardiac index; RAP, right atrial pressure; AUC, area under the curve.
(TIF)

S1 File Analysis data. (XLSX)

## References

[1] Forrester JS, Diamond G, Chatterjee K, Swan HJ. Medical therapy of acute myocardial infarction by application of hemodynamic subsets (first of two parts). N Engl J Med. 1976;295(24):1356–62. doi: 10.1056/NEJM197612092952406 790191

[2] Forrester JS, Diamond G, Chatterjee K, Swan HJ. Medical therapy of acute myocardial infarction by application of hemodynamic subsets (second of two parts). N Engl J Med. 1976;295(25):1404–13. doi: 10.1056/NEJM197612162952505 790194

[3] Binanay C, Califf RM, Hasselblad V, O’Connor CM, Shah MR, Sopko G, et al. Evaluation study of congestive heart failure and pulmonary artery catheterization effectiveness: the ESCAPE trial. JAMA. 2005;294(13):1625–33. doi: 10.1001/jama.294.13.1625 16204662

[4] Nickel N, Golpon H, Greer M, Knudsen L, Olsson K, Westerkamp V, et al. The prognostic impact of follow-up assessments in patients with idiopathic pulmonary arterial hypertension. Eur Respir J. 2012;39(3):589–96. doi: 10.1183/09031936.00092311 21885392

[5] Drazner MH, Hamilton MA, Fonarow G, Creaser J, Flavell C, Stevenson LW. Relationship between right and left-sided filling pressures in 1000 patients with advanced heart failure. J Heart Lung Transplant. 1999;18(11):1126–32. doi: 10.1016/s1053-2498(99)00070-4 10598737

[6] Drazner MH, Brown RN, Kaiser PA, Cabuay B, Lewis NP, Semigran MJ, et al. Relationship of right- and left-sided filling pressures in patients with advanced heart failure: a 14-year multi-institutional analysis. J Heart Lung Transplant. 2012;31(1):67–72. doi: 10.1016/j.healun.2011.09.003 22071240

[7] Padang R, Chandrashekar N, Indrabhinduwat M, Scott CG, Luis SA, Chandrasekaran K, et al. Aetiology and outcomes of severe right ventricular dysfunction. Eur Heart J. 2020;41(12):1273–82. doi: 10.1093/eurheartj/ehaa037 32047900

[8] Galie N, Humbert M, Vachiery JL, Gibbs S, Lang I, Torbicki A, et al. 2015 ESC/ERS Guidelines for the diagnosis and treatment of pulmonary hypertension: The Joint Task Force for the Diagnosis and Treatment of Pulmonary Hypertension of the European Society of Cardiology (ESC) and the European Respiratory Society (ERS): Endorsed by: Association for European Paediatric and Congenital Cardiology (AEPC), International Society for Heart and Lung Transplantation (ISHLT). Eur Heart J. 2016;37(1):67–119. doi: 10.1093/eurheartj/ehv317 26320113

[9] Boucly A, Weatherald J, Savale L, Jais X, Cottin V, Prevot G, et al. Risk assessment, prognosis and guideline implementation in pulmonary arterial hypertension. Eur Respir J. 2017;50(2). doi: 10.1183/13993003.00889-2017 28775050

[10] Ibe T, Wada H, Sakakura K, Ikeda N, Yamada Y, Sugawara Y, et al. Pulmonary hypertension due to left heart disease: The prognostic implications of diastolic pulmonary vascular pressure gradient. J Cardiol. 2016;67(6):555–9. doi: 10.1016/j.jjcc.2015.07.015 26299611

[11] Kovacs G, Avian A, Pienn M, Naeije R, Olschewski H. Reading pulmonary vascular pressure tracings. How to handle the problems of zero leveling and respiratory swings. Am J Respir Crit Care Med. 2014;190(3):252–7. doi: 10.1164/rccm.201402-0269PP 24869464

[12] Ponikowski P, Voors AA, Anker SD, Bueno H, Cleland JG, Coats AJ, et al. 2016 ESC Guidelines for the diagnosis and treatment of acute and chronic heart failure: The Task Force for the diagnosis and treatment of acute and chronic heart failure of the European Society of Cardiology (ESC). Developed with the special contribution of the Heart Failure Association (HFA) of the ESC. Eur J Heart Fail. 2016;18(8):891–975. doi: 10.1002/ejhf.592 27207191

[13] Sakakura K, Kubo N, Ako J, Wada H, Fujiwara N, Funayama H, et al. Peak C-reactive protein level predicts long-term outcomes in type B acute aortic dissection. Hypertension. 2010;55(2):422–9. doi: 10.1161/HYPERTENSIONAHA.109.143131 20038755

[14] Yamanaka H, Japanese Society of G, Nucleic Acid M. Japanese guideline for the management of hyperuricemia and gout: second edition. Nucleosides Nucleotides Nucleic Acids. 2011;30(12):1018–29. doi: 10.1080/15257770.2011.596496 22132951

[15] Nutritional anaemias. Report of a WHO scientific group. World Health Organ Tech Rep Ser. 1968;405:5–37. 4975372

[16] Imai E, Horio M, Nitta K, Yamagata K, Iseki K, Tsukamoto Y, et al. Modification of the Modification of Diet in Renal Disease (MDRD) Study equation for Japan. Am J Kidney Dis. 2007;50(6):927–37. doi: 10.1053/j.ajkd.2007.09.004 18037093

[17] Rudski LG, Lai WW, Afilalo J, Hua L, Handschumacher MD, Chandrasekaran K, et al. Guidelines for the echocardiographic assessment of the right heart in adults: a report from the American Society of Echocardiography endorsed by the European Association of Echocardiography, a registered branch of the European Society of Cardiology, and the Canadian Society of Echocardiography. J Am Soc Echocardiogr. 2010;23(7):685–713; quiz 86–8. doi: 10.1016/j.echo.2010.05.010 20620859

[18] Hamaguchi S, Kinugawa S, Goto D, Tsuchihashi-Makaya M, Yokota T, Yamada S, et al. Predictors of long-term adverse outcomes in elderly patients over 80 years hospitalized with heart failure.—A report from the Japanese Cardiac Registry of Heart Failure in Cardiology (JCARE-CARD). Circ J. 2011;75(10):2403–10. doi: 10.1253/circj.cj-11-0267 21778592

[19] Ho KK, Pinsky JL, Kannel WB, Levy D. The epidemiology of heart failure: the Framingham Study. J Am Coll Cardiol. 1993;22(4 Suppl A):6A–13A. doi: 10.1016/0735-1097(93)90455-a 8376698

[20] Kenchaiah S, Pocock SJ, Wang D, Finn PV, Zornoff LA, Skali H, et al. Body mass index and prognosis in patients with chronic heart failure: insights from the Candesartan in Heart failure: Assessment of Reduction in Mortality and morbidity (CHARM) program. Circulation. 2007;116(6):627–36. doi: 10.1161/CIRCULATIONAHA.106.679779 17638930

[21] Groenveld HF, Januzzi JL, Damman K, van Wijngaarden J, Hillege HL, van Veldhuisen DJ, et al. Anemia and mortality in heart failure patients a systematic review and meta-analysis. J Am Coll Cardiol. 2008;52(10):818–27. doi: 10.1016/j.jacc.2008.04.061 18755344

[22] Yamauchi T, Sakata Y, Miura M, Onose T, Tsuji K, Abe R, et al. Prognostic Impact of Atrial Fibrillation and New Risk Score of Its Onset in Patients at High Risk of Heart Failure- A Report From the CHART-2 Study. Circ J. 2017;81(2):185–94. doi: 10.1253/circj.CJ-16-0759 28090009

[23] Anker SD, Doehner W, Rauchhaus M, Sharma R, Francis D, Knosalla C, et al. Uric acid and survival in chronic heart failure: validation and application in metabolic, functional, and hemodynamic staging. Circulation. 2003;107(15):1991–7. doi: 10.1161/01.CIR.0000065637.10517.A0 12707250

[24] McAlister FA, Ezekowitz J, Tonelli M, Armstrong PW. Renal insufficiency and heart failure: prognostic and therapeutic implications from a prospective cohort study. Circulation. 2004;109(8):1004–9. doi: 10.1161/01.CIR.0000116764.53225.A9 14769700

[25] Vedin O, Lam CSP, Koh AS, Benson L, Teng THK, Tay WT, et al. Significance of Ischemic Heart Disease in Patients With Heart Failure and Preserved, Midrange, and Reduced Ejection Fraction: A Nationwide Cohort Study. Circ Heart Fail. 2017;10(6). 10.1161/CIRCHEARTFAILURE.117.003875 28615366

[26] Cho JH, Choe WS, Cho HJ, Lee HY, Jang J, Lee SE, et al. Comparison of Characteristics and 3-Year Outcomes in Patients With Acute Heart Failure With Preserved, Mid-Range, and Reduced Ejection Fraction. Circ J. 2019;83(2):347–56. doi: 10.1253/circj.CJ-18-0543 30404976

[27] Meta-analysis Global Group in Chronic Heart F. The survival of patients with heart failure with preserved or reduced left ventricular ejection fraction: an individual patient data meta-analysis. Eur Heart J. 2012;33(14):1750–7. doi: 10.1093/eurheartj/ehr254 21821849

[28] Lauritsen J, Gustafsson F, Abdulla J. Characteristics and long-term prognosis of patients with heart failure and mid-range ejection fraction compared with reduced and preserved ejection fraction: a systematic review and meta-analysis. ESC Heart Fail. 2018;5(4):685–94. doi: 10.1002/ehf2.12283 29660263 PMC6073025

[29] Altaie S, Khalife W. The prognosis of mid-range ejection fraction heart failure: a systematic review and meta-analysis. ESC Heart Fail. 2018;5(6):1008–16. doi: 10.1002/ehf2.12353 30211480 PMC6301154

[30] Miura M, Sugimura K, Sakata Y, Miyata S, Tadaki S, Yamauchi T, et al. Prognostic Impact of Loop Diuretics in Patients With Chronic Heart Failure- Effects of Addition of Renin-Angiotensin-Aldosterone System Inhibitors and beta-Blockers. Circ J. 2016;80(6):1396–403. doi: 10.1253/circj.CJ-16-0216 27170200

[31] Cooper LB, Mentz RJ, Stevens SR, Felker GM, Lombardi C, Metra M, et al. Hemodynamic Predictors of Heart Failure Morbidity and Mortality: Fluid or Flow? J Card Fail. 2016;22(3):182–9. doi: 10.1016/j.cardfail.2015.11.012 26703245 PMC4779722

[32] Patel CB, DeVore AD, Felker GM, Wojdyla DM, Hernandez AF, Milano CA, et al. Characteristics and outcomes of patients with heart failure and discordant findings by right-sided heart catheterization and cardiopulmonary exercise testing. Am J Cardiol. 2014;114(7):1059–64. doi: 10.1016/j.amjcard.2014.07.022 25212547

[33] Nohria A, Tsang SW, Fang JC, Lewis EF, Jarcho JA, Mudge GH, et al. Clinical assessment identifies hemodynamic profiles that predict outcomes in patients admitted with heart failure. J Am Coll Cardiol. 2003;41(10):1797–804. doi: 10.1016/s0735-1097(03)00309-7 12767667

[34] Damman K, Navis G, Smilde TD, Voors AA, van der Bij W, van Veldhuisen DJ, et al. Decreased cardiac output, venous congestion and the association with renal impairment in patients with cardiac dysfunction. Eur J Heart Fail. 2007;9(9):872–8. doi: 10.1016/j.ejheart.2007.05.010 17586090

[35] Jacob R, Dierberger B, Kissling G. Functional significance of the Frank-Starling mechanism under physiological and pathophysiological conditions. Eur Heart J. 1992;13 Suppl E:7–14. doi: 10.1093/eurheartj/13.suppl_e.7 1478214

[36] Gardner RS, Henderson G, McDonagh TA. The prognostic use of right heart catheterization data in patients with advanced heart failure: how relevant are invasive procedures in the risk stratification of advanced heart failure in the era of neurohormones? J Heart Lung Transplant. 2005;24(3):303–9. doi: 10.1016/j.healun.2004.01.010 15737757

