# Supplementary material for: Cardiac index predicts long-term outcomes in patients with heart failure
Source: PLoS One. 2021 Jun 4;16(6):e0252833. doi: 10.1371/journal.pone.0252833 (PMC8177638; doi:10.1371/journal.pone.0252833)
Supplement: S1 Table — (DOCX) [file pone.0252833.s001.docx]

S1 Table. Multivariate Cox regression analysis predicting cardiac death.

| Variables | Model 1 | | |  | Model 2 | | |  | Model 3 | | |
| --- | --- | --- | --- | --- | --- | --- | --- | --- | --- | --- | --- |
|  | HR | 95% CI | *P* value |  | HR | 95% CI | *P* value |  | HR | 95% CI | *P* value |
| Hemodynamic categorization of heart failure |  |  |  |  |  |  |  |  |  |  |  |
| PRE-CI/H-RAP (vs. PRE-CI/L-RAP) | 1.32 | 0.59 - 2.97 | 0.51 |  |  |  |  |  |  |  |  |
| RED-CI/L-RAP (vs. PRE-CI/L-RAP) | 2.56 | 1.07 - 6.08 | 0.03 |  |  |  |  |  |  |  |  |
| RED-CI/H-RAP (vs. PRE-CI/L-RAP) | 4.11 | 1.84 - 9.20 | 0.001 |  |  |  |  |  |  |  |  |
| Hemodynamic categorization of heart failure |  |  |  |  |  |  |  |  |  |  |  |
| PRE-CI/L-RAP (vs. PRE-CI/H-RAP) |  |  |  |  | 0.76 | 0.34 - 1.71 | 0.51 |  |  |  |  |
| RED-CI/L-RAP (vs. PRE-CI/H-RAP) |  |  |  |  | 1.94 | 0.82 - 4.60 | 0.13 |  |  |  |  |
| RED-CI/H-RAP (vs. PRE-CI/H-RAP) |  |  |  |  | 3.12 | 1.54 - 6.33 | 0.002 |  |  |  |  |
| Hemodynamic categorization of heart failure |  |  |  |  |  |  |  |  |  |  |  |
| PRE-CI/L-RAP (vs. RED-CI/L-RAP) |  |  |  |  |  |  |  |  | 0.39 | 0.17 - 0.93 | 0.03 |
| PRE-CI/H-RAP (vs. RED-CI/L-RAP) |  |  |  |  |  |  |  |  | 0.52 | 0.22 - 1.23 | 0.13 |
| RED-CI/H-RAP (vs. RED-CI/L-RAP) |  |  |  |  |  |  |  |  | 1.61 | 0.73 - 3.57 | 0.24 |
| mPAP >20 mmHg | 1.02 | 0.46 - 2.26 | 0.97 |  | 1.02 | 0.46 - 2.26 | 0.97 |  | 1.02 | 0.46 - 2.26 | 0.97 |
| PAWP ≥18 mmHg | 1.60 | 0.79 - 3.23 | 0.19 |  | 1.60 | 0.79 - 3.23 | 0.19 |  | 1.60 | 0.79 - 3.23 | 0.19 |
| Age (10 year increase) | 1.17 | 0.92 - 1.49 | 0.21 |  | 1.17 | 0.92 - 1.49 | 0.21 |  | 1.17 | 0.92 - 1.49 | 0.21 |
| Male sex (vs. female) | 0.71 | 0.39 - 1.26 | 0.24 |  | 0.71 | 0.39 - 1.26 | 0.24 |  | 0.71 | 0.39 - 1.26 | 0.24 |
| Overweight (BMI ≥25 kg/m^2^) | 0.63 | 0.34 - 1.18 | 0.15 |  | 0.63 | 0.34 - 1.18 | 0.15 |  | 0.63 | 0.34 - 1.18 | 0.15 |
| Anemia | 1.75 | 0.99 - 3.09 | 0.05 |  | 1.75 | 0.99 - 3.09 | 0.05 |  | 1.75 | 0.99 - 3.09 | 0.05 |
| Atrial fibrillation or flutter | 0.89 | 0.50 - 1.61 | 0.70 |  | 0.89 | 0.50 - 1.61 | 0.70 |  | 0.89 | 0.50 - 1.61 | 0.70 |
| Hyperuricemia | 1.07 | 0.59 - 1.97 | 0.82 |  | 1.07 | 0.59 - 1.97 | 0.82 |  | 1.07 | 0.59 - 1.97 | 0.82 |
| Impaired renal function | 0.98 | 0.55 - 1.74 | 0.93 |  | 0.98 | 0.55 - 1.74 | 0.93 |  | 0.98 | 0.55 - 1.74 | 0.93 |
| Ischemic heart disease | 2.46 | 1.31 - 4.64 | 0.005 |  | 2.46 | 1.31 - 4.64 | 0.005 |  | 2.46 | 1.31 - 4.64 | 0.005 |
| Loop diuretic use | 0.76 | 0.42 - 1.87 | 0.84 |  | 0.76 | 0.42 - 1.87 | 0.84 |  | 0.76 | 0.42 - 1.87 | 0.84 |
| Categorization of LVEF |  |  |  |  |  |  |  |  |  |  |  |
| Reduced LVEF (vs. preserved LVEF) | 1.28 | 0.63 - 2.59 | 0.49 |  | 1.28 | 0.63 - 2.59 | 0.49 |  | 1.28 | 0.63 - 2.59 | 0.49 |
| Mid-range LVEF (vs. preserved LVEF) | 1.10 | 0.44 - 2.77 | 0.84 |  | 1.10 | 0.44 - 2.77 | 0.84 |  | 1.10 | 0.44 - 2.77 | 0.84 |

HR, hazard ratio; CI, confidence interval; mPAP, mean pulmonary artery pressure; PAWP, pulmonary artery wedge pressure; BMI, body mass index; LVEF, left ventricular ejection fraction.
