# Supplementary material for: Cardiac index predicts long-term outcomes in patients with heart failure
Source: PLoS One. 2021 Jun 4;16(6):e0252833. doi: 10.1371/journal.pone.0252833 (PMC8177638; doi:10.1371/journal.pone.0252833)
Supplement: S2 Table — (DOCX) [file pone.0252833.s002.docx]

S2 Table. Multivariate Cox regression analysis predicting heart failure readmission.

| Variables | Model 1 | | |  | Model 2 | | |  | Model 3 | | |
| --- | --- | --- | --- | --- | --- | --- | --- | --- | --- | --- | --- |
|  | HR | 95% CI | *P* value |  | HR | 95% CI | *P* value |  | HR | 95% CI | *P* value |
| Hemodynamic categorization of heart failure |  |  |  |  |  |  |  |  |  |  |  |
| PRE-CI/H-RAP (vs. PRE-CI/L-RAP) | 0.94 | 0.59 - 1.51 | 0.79 |  |  |  |  |  |  |  |  |
| RED-CI/L-RAP (vs. PRE-CI/L-RAP) | 2.12 | 1.30 - 3.46 | 0.003 |  |  |  |  |  |  |  |  |
| RED-CI/H-RAP (vs. PRE-CI/L-RAP) | 1.91 | 1.15 - 3.18 | 0.01 |  |  |  |  |  |  |  |  |
| Hemodynamic categorization of heart failure |  |  |  |  |  |  |  |  |  |  |  |
| PRE-CI/L-RAP (vs. PRE-CI/H-RAP) |  |  |  |  | 1.07 | 0.66 - 1.71 | 0.79 |  |  |  |  |
| RED-CI/L-RAP (vs. PRE-CI/H-RAP) |  |  |  |  | 2.26 | 1.35 - 3.79 | 0.002 |  |  |  |  |
| RED-CI/H-RAP (vs. PRE-CI/H-RAP) |  |  |  |  | 2.04 | 1.27 - 3.26 | 0.003 |  |  |  |  |
| Hemodynamic categorization of heart failure |  |  |  |  |  |  |  |  |  |  |  |
| PRE-CI/L-RAP (vs. RED-CI/L-RAP) |  |  |  |  |  |  |  |  | 0.47 | 0.29 - 0.77 | 0.003 |
| PRE-CI/H-RAP (vs. RED-CI/L-RAP) |  |  |  |  |  |  |  |  | 0.44 | 0.26 - 0.74 | 0.002 |
| RED-CI/H-RAP (vs. RED-CI/L-RAP) |  |  |  |  |  |  |  |  | 0.90 | 0.54 - 1.50 | 0.69 |
| mPAP >20 mmHg | 1.21 | 0.77 - 1.91 | 0.41 |  | 1.21 | 0.77 - 1.91 | 0.41 |  | 1.21 | 0.77 - 1.91 | 0.41 |
| PAWP ≥18 mmHg | 1.27 | 0.83 - 1.93 | 0.27 |  | 1.27 | 0.83 - 1.93 | 0.27 |  | 1.27 | 0.83 - 1.93 | 0.27 |
| Age (10 year increase) | 1.24 | 1.06 - 1.45 | 0.006 |  | 1.24 | 1.06 - 1.45 | 0.006 |  | 1.24 | 1.06 - 1.45 | 0.006 |
| Male sex (vs. female) | 0.81 | 0.56 - 1.15 | 0.24 |  | 0.81 | 0.56 - 1.15 | 0.24 |  | 0.81 | 0.56 - 1.15 | 0.24 |
| Overweight (BMI ≥25 kg/m^2^) | 1.01 | 0.70 - 1.45 | 0.98 |  | 1.01 | 0.70 - 1.45 | 0.98 |  | 1.01 | 0.70 - 1.45 | 0.98 |
| Anemia | 1.58 | 1.11 - 2.26 | 0.01 |  | 1.58 | 1.11 - 2.26 | 0.01 |  | 1.58 | 1.11 - 2.26 | 0.01 |
| Atrial fibrillation or flutter | 1.03 | 0.73 - 1.46 | 0.87 |  | 1.03 | 0.73 - 1.46 | 0.87 |  | 1.03 | 0.73 - 1.46 | 0.87 |
| Hyperuricemia | 0.87 | 0.60 - 1.26 | 0.46 |  | 0.87 | 0.60 - 1.26 | 0.46 |  | 0.87 | 0.60 - 1.26 | 0.46 |
| Impaired renal function | 1.21 | 0.85 - 1.73 | 0.29 |  | 1.21 | 0.85 - 1.73 | 0.29 |  | 1.21 | 0.85 - 1.73 | 0.29 |
| Ischemic heart disease | 1.69 | 1.09 - 2.62 | 0.02 |  | 1.69 | 1.09 - 2.62 | 0.02 |  | 1.69 | 1.09 - 2.62 | 0.02 |
| Loop diuretic use | 1.00 | 0.62 - 1.62 | 1.00 |  | 1.00 | 0.62 - 1.62 | 1.00 |  | 1.00 | 0.62 - 1.62 | 1.00 |
| Categorization of LVEF |  |  |  |  |  |  |  |  |  |  |  |
| Reduced LVEF (vs. preserved LVEF) | 1.33 | 0.89 - 2.00 | 0.17 |  | 1.33 | 0.89 - 2.00 | 0.17 |  | 1.33 | 0.89 - 2.00 | 0.17 |
| Mid-range LVEF (vs. preserved LVEF) | 0.57 | 0.30 - 1.11 | 0.10 |  | 0.57 | 0.30 - 1.11 | 0.10 |  | 0.57 | 0.30 - 1.11 | 0.10 |

HR, hazard ratio; CI, confidence interval; mPAP, mean pulmonary artery pressure; PAWP, pulmonary artery wedge pressure; BMI, body mass index; LVEF, left ventricular ejection fraction.
