# Supplementary figures and images for: Cardiac index predicts long-term outcomes in patients with heart failure
Source: PLoS One. 2021 Jun 4;16(6):e0252833. doi: 10.1371/journal.pone.0252833 (PMC8177638; doi:10.1371/journal.pone.0252833)

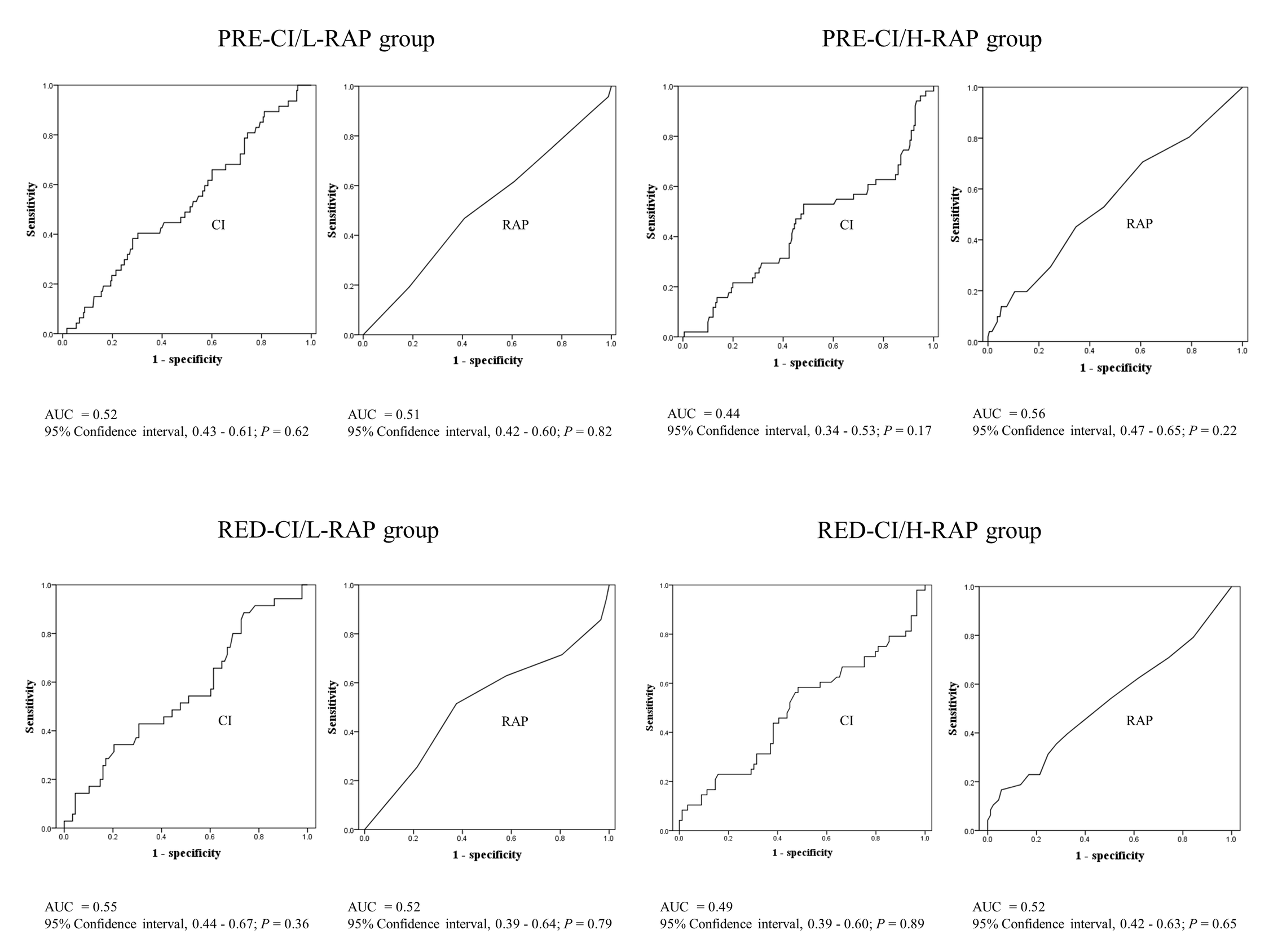

Supplement: S1 Fig — CI, cardiac index; RAP, right atrial pressure; AUC, area under the curve. (TIF) [file pone.0252833.s003.tif]
